# Not Your Typical Thunderclap Headache

**DOI:** 10.7759/cureus.37955

**Published:** 2023-04-21

**Authors:** Ivan Ivanov, Dimitri Livshits, Brenda Sokup, Angela Frisby, Nirav Patel

**Affiliations:** 1 Emergency Medicine, New York City Health + Hospitals/South Brooklyn Health, Brooklyn, USA

**Keywords:** subarachnoid hemorrhage, thunderclap, headache, neurosurgery, emergency medicine

## Abstract

We present the case of a young adult female who presented to the emergency department with headache and vomiting. After treatment with intravenous fluids, diphenhydramine and metoclopramide the headache completely resolved. Because of the patient's persistent symptoms and past medical history of systemic lupus erythematosus, a noncontrast head CT scan was done. In this case, the patient had a subarachnoid hemorrhage with edema and mass effect, detected on a noncontrast head CT scan. The patient required a nicardipine drip for blood pressure control. The patient recovered well and was discharged at her normal state of health. This case demonstrates the importance of maintaining high clinical suspicion for life-threatening emergencies even in patients with unremarkable physical exams who experience symptomatic improvement after treatment.

## Introduction

Headache is a common complaint in the emergency department, accounting for 2% of all visits [1]. Most patients with a subarachnoid hemorrhage present with a sudden onset headache of maximal intensity, associated with nausea and vomiting, and describe the headache as the worst headache they have ever experienced [1]. This case report is a unique presentation of a significant subarachnoid hemorrhage in a young adult female who did not exhibit the usual symptoms of a subarachnoid hemorrhage. The patient did not identify the headache as the “worst headache of her life,” and the headache improved symptomatically with treatment. Due to the patient's comorbidities and chronicity of symptoms, a noncontrast head CT scan was ordered and was diagnostic. This unique case presentation is a reminder to always have high suspicion for life-threatening conditions despite symptomatic improvement, and to escalate diagnostic care when indicated.

## Case presentation

A 22-year-old female with a past medical history of systemic lupus erythematosus and seizure disorder presented to the emergency department with occipital headache for three hours. She reported a fall three months prior which was caused by a seizure. Since her fall she reported similar daily throbbing headaches, difficulty concentrating, and intermittent nausea and vomiting. 

Vital signs and the physical examination were unremarkable on presentation. The patient was given one liter lactated ringers, 50 mg diphenhydramine, and 10 mg metoclopramide intravenously which completely resolved her symptoms.

**Figure 1:**
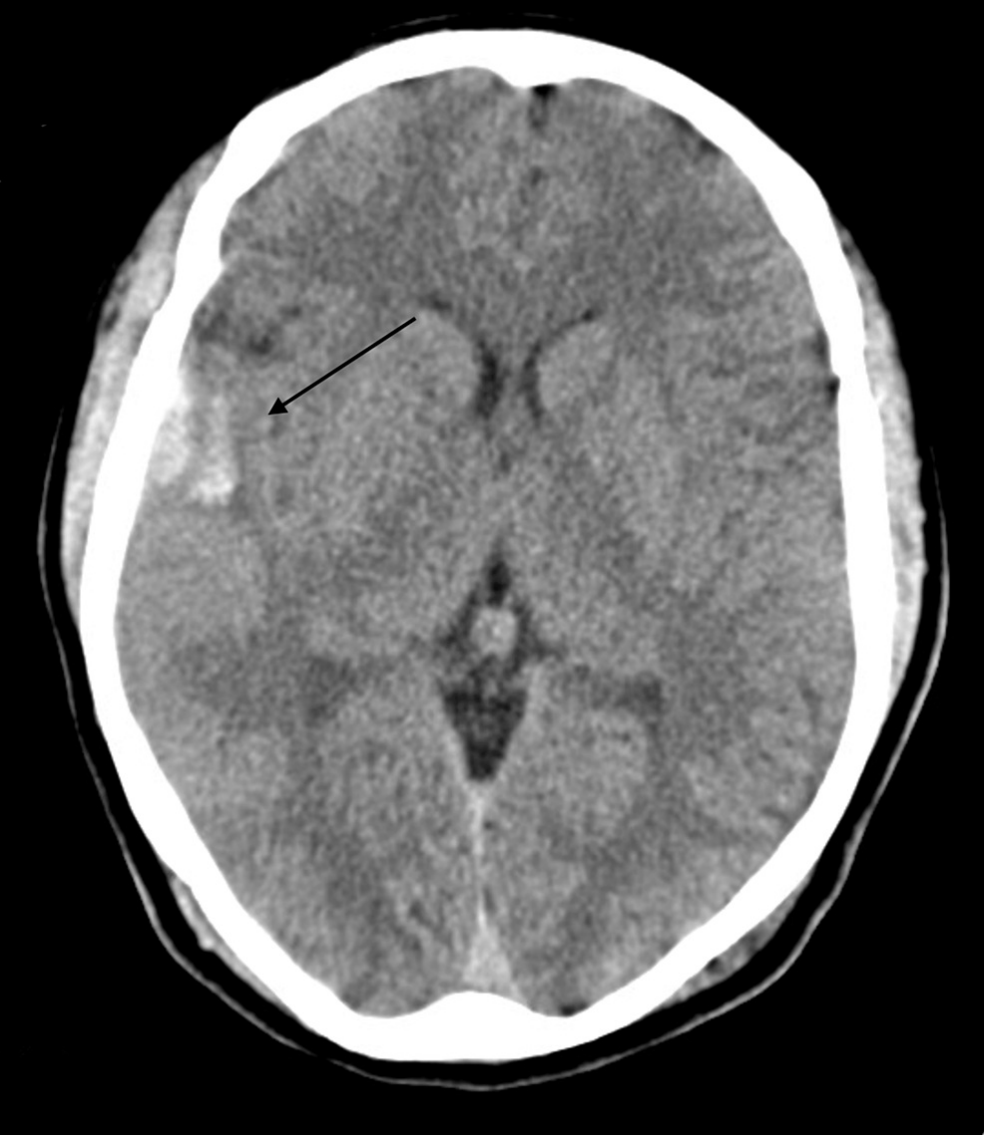
Subarachnoid hemorrhage in the right frontotemporal region associated with edema and mass effect.

**Figure 2:**
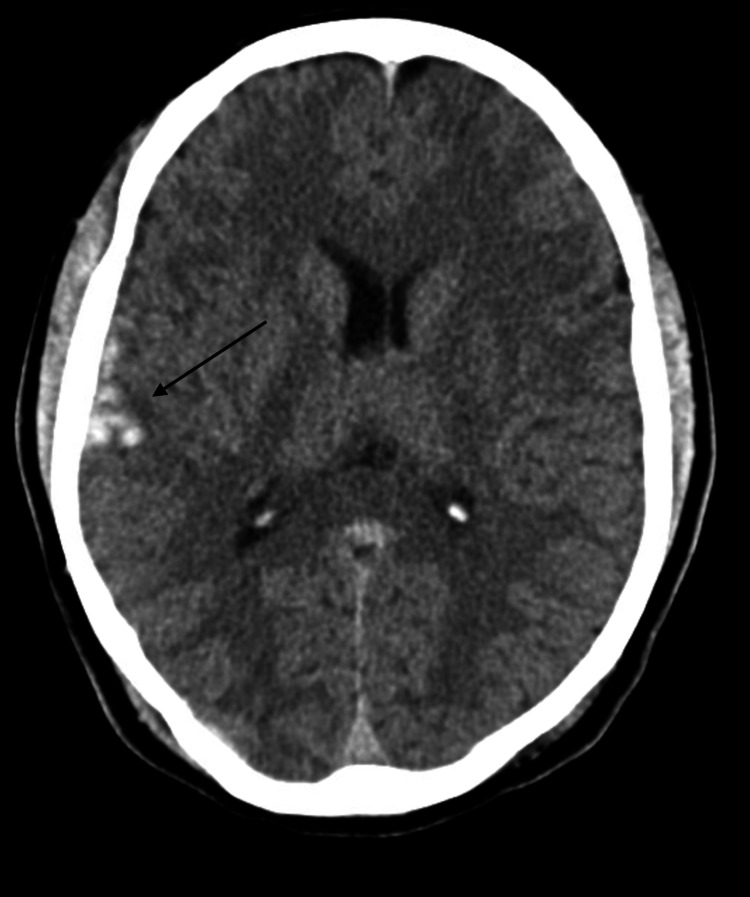
Subarachnoid hemorrhage in the right frontotemporal region associated with edema and mass effect.

The patient reported that after her fall three months prior, imaging was done at an outside facility which was negative. The repeat neurological exam was again unremarkable. Given her past medical history of systemic lupus erythematosus, seizure disorder, and remote history of trauma, a noncontrast CT head was performed and revealed a subarachnoid hemorrhage with edema and mass effect (Figures 1, 2).

During her time in the emergency department her blood pressure began to rise and she was started on a nicardipine drip which controlled her blood pressure to below a systolic of 150 mmHg. CT angiogram of the brain was obtained to rule out cerebral aneurysm or dissection because they are strongly associated with systemic lupus erythematosus [1]. CT angiogram was obtained and was negative for aneurysm or dissection. The patient was admitted to the neurosurgical intensive care unit. Six hours later a repeat noncontrast head CT showed an unchanged subarachnoid hemorrhage. Over the next three days the patient was titrated off the nicardipine drip and started on 5 mg oral amlodipine. Ultimately, the patient did well and was discharged in her normal state of health. 

## Discussion

The classic presentation of a subarachnoid hemorrhage is a severe, sudden onset headache which is often described as “the worst headache” the patient ever had [1]. Ruptured aneurysms are responsible for 85% of all non-traumatic subarachnoid hemorrhages [2]. The pain reaches maximum intensity in one minute after onset [3]. This is known as a “thunderclap” headache. This characterization in combination with focal neurological findings, seizure, or altered level of consciousness can also be caused by intracerebral hemorrhage, reversible cerebral vasoconstriction syndrome, posterior reversible encephalopathy syndrome, hypertensive crisis, cervical artery dissection, or ischemic stroke [3]. With respect to subarachnoid hemorrhage, headache is often an isolated finding [4]. 

With aneurysmal rupture and hemorrhage, the risk of rebleeding is highest in the first 24 hours, blood pressure management is key [5]. Nicardipine and labetalol can be used and titrated to a systolic blood pressure of less than 150 mmHg [6]. Vasospasm is most common in two days to three weeks after subarachnoid hemorrhage [7]. Nimodipine should be started within the first four days unless contraindicated [8]. To prevent further delayed cerebral ischemia hyperglycemia, hyperthermia, and hypothermia should be treated. 

Subarachnoid hemorrhage is the most common cause of “thunderclap” headache [1]. If the patient presents within six hours of presentation, a noncontrast head CT is 100% sensitive [1]. If the presentation is delayed, a lumbar puncture or CT angiogram should be considered [1].

## Conclusions

Subarachnoid hemorrhage is the most common cause of a “thunderclap” headache, with associated symptoms including nausea, vomiting, seizures, and loss of consciousness. Subarachnoid hemorrhage should not be ruled out based on symptomatic improvement or an unremarkable neurological exam. If the patient has concerning comorbidities, red flag symptoms, or a worrisome history of presenting illness then imaging is critical to rule out life-threatening pathology. Maintaining a high index of suspicion is key to making the diagnosis. Definitive diagnosis is made by noncontrast head CT, lumbar puncture or CT angiogram. Once subarachnoid hemorrhage is diagnosed, blood pressure control is key to reducing the chance of rebleeding.
